# Inhibiting Methanogenesis Stimulated de novo Synthesis of Microbial Amino Acids in Mixed Rumen Batch Cultures Growing on Starch but not on Cellulose

**DOI:** 10.3390/microorganisms8060799

**Published:** 2020-05-26

**Authors:** Emilio M. Ungerfeld, M. Fernanda Aedo, Camila Muñoz, Natalie L. Urrutia, Emilio D. Martínez, Marcelo Saldivia

**Affiliations:** 1 Centro Regional de Investigación Carillanca, Instituto de Investigaciones Agropecuarias INIA, Temuco 4880000, Chile; aedo.enriquez13@gmail.com; 2 Centro Regional de Investigación Remehue, Instituto de Investigaciones Agropecuarias INIA, Temuco 5290000, Chile; camila.munoz@inia.cl (C.M.); natalie.urrutia@inia.cl (N.L.U.); 3 Facultad de Ciencias Veterinarias, Universidad Austral de Chile, Valdivia 5090000, Chile; emiliomartinez@uach.cl (E.D.M.); marcelosaldiviamv@gmail.com (M.S.)

**Keywords:** rumen, fermentation, methanogenesis, metabolic hydrogen, amino acids, cellulose, starch

## Abstract

Ameliorating methane (CH_4_) emissions from ruminants would have environmental benefits, but it is necessary to redirect metabolic hydrogen ([H]) toward useful sinks to also benefit animal productivity. We hypothesized that inhibiting rumen methanogenesis would increase de novo synthesis of microbial amino acids (AA) as an alternative [H] sink if sufficient energy and carbon are provided. We examined the effects of inhibiting methanogenesis with 9, 10-anthraquione (AQ) on mixed rumen batch cultures growing on cellulose or starch as sources of energy and carbon contrasting in fermentability, with ammonium (NH_4_^+^) or trypticase (Try) as nitrogen (N) sources. Inhibiting methanogenesis with AQ inhibited digestion with cellulose but not with starch, and decreased propionate and increased butyrate molar percentages with both substrates. Inhibiting methanogenesis with 9, 10-anthraquinone increased de novo synthesis of microbial AA with starch but not with cellulose. The decrease in the recovery of [H] caused by the inhibition of methanogenesis was more moderate with starch due to an enhancement of butyrate and AA as [H] sinks. There may be an opportunity to simultaneously decrease the emissions of CH_4_ and N with some ruminant diets and replace plant protein supplements with less expensive non-protein nitrogen sources such as urea.

## 1. Introduction

The abatement of methane (CH_4_) emissions is key to achieve a rapid mitigation in the emission of greenhouse gases from anthropogenic origin, because CH_4_, having a shorter atmospheric lifetime than CO_2_, has an 85-fold Global Warming Potential within a 20-yr timeframe [1,2]. Enteric CH_4_ formed in the rumen of domestic ruminants is a major source of anthropogenic CH_4_ [3]. Therefore, decreasing rumen methanogenesis has become an important target to achieve a short term decrease in the emissions of anthropogenic greenhouse gases.

Chemical inhibitors of methanogenesis are the most effective strategy to decrease CH_4_ production in the rumen [4]. However, the incorporation of inhibitors of methanogenesis as feed additives in ruminant diets would increase feed costs. Producers are unlikely to increase the costs of animal feed unless inhibiting rumen methanogenesis can enhance animal productivity. The release to the atmosphere of CH_4_ produced in the rumen is an energy inefficiency, and decreasing energy losses such as CH_4_ is a potential means to increase ruminant productivity [5,6]. So far, however, the potential of translating energy saved in CH_4_ not formed in the rumen into greater animal productivity has not been consistently achieved, with benefits observed in some, but not in all, studies [7].

A poorly understood consequence of the inhibition of rumen methanogenesis observed in in vitro experiments is a consistent and severe decline in the recovery of metabolic hydrogen ([H]), quantified as reducing equivalents pairs ([2H]), in volatile fatty acids (VFA), CH_4_ and dihydrogen (H_2_) [8]. It is important to identify the unaccounted sinks of [H] that become important when CH_4_ formation in the rumen is inhibited because some of those [H] sinks may be of nutritional value whilst others may not. Furthermore, the shifts in [H] sinks occurring when CH_4_ production is inhibited may require animal diets to be formulated considering changes in the flows of absorbed nutrients caused by the methanogenesis inhibition intervention [7].

The inhibition of methanogenesis in rumen batch cultures has been shown to inhibit deamination of amino acids (AA) [9,10], an effect that can be mediated by an increase in the intracellular NADH to NAD^+^ ratio [11]. Deamination releases one mole of [2H] per mole of ammonia (NH_3_) released. On the other hand, amination, the incorporation of NH_3_ into carbon chains in AA synthesis, incorporates one mole of [2H], donated by NADH or NADPH, per mole of NH_3_ incorporated [12,13]. It is then conceivable that an increase in the intracellular NADH to NAD^+^ ratio occurring when methanogenesis is inhibited in rumen fermentation could stimulate the incorporation of NH_3_ into carbon chains and the synthesis of AA [8]. Consequences of inhibiting rumen methanogenesis on microbial protein synthesis can be of importance because microbial protein produced in the rumen is the main [13,14] and most economical source of absorbed AA for ruminants, and ammonium (NH_4_^+^), the predominant protonated species of NH_3_ at rumen pH, is a major source of nitrogen (N) for microbial protein synthesis [15]. However, the hypothesis that inhibiting methanogenesis would increase the synthesis of microbial AA, was not confirmed when cellulose was incubated with eight different inhibitors of methanogenesis in mixed rumen batch cultures [16].

It is possible that an increase in the availability of [2H] caused by inhibiting methanogenesis did not result in a response in the incorporation of NH_3_ into carbon chains if amination became limited by the availability of carbon precursors or by ATP in batch cultures fermenting cellulose [16]. A more rapidly degradable substrate such as starch may provide carbon and energy for AA synthesis at faster rates. We hypothesized that the response to methanogenesis inhibition in the synthesis of microbial AA would be greater with starch than with cellulose. Our objective was to compare the effects of inhibiting methanogenesis in rumen batch cultures with cellulose or starch as energy and carbon substrates on fermentation, the production of microbial biomass and AA, and [2H] balance. We concluded that inhibiting methanogenesis stimulated AA synthesis with starch but not with cellulose. We also found that the decrease in the recovery of [2H] when methanogenesis was inhibited was less pronounced with starch compared to cellulose, explained largely by greater recovery of [2H] in butyrate production and AA synthesis.

## 2. Materials and Methods

All animal procedures in Project Fondecyt 1160764 were approved by the Comité de Ética para el Uso de Animales en Investigación, Instituto de Investigaciones Agropecuarias INIA, Approval number 02/2016, from 7 June 2016.

### 2.1. Treatments and Incubations

The experiment was designed as a 2 × 2 × 2 arrangement of treatments, with the three factors being: i) Methanogenesis inhibition [M factor, comprising Control (AQ-) or 9, 10-Anthraquinone (AQ+) levels], ii) Energy and carbon source [E factor, comprising Cellulose (C) or Starch (S) levels], and iii) N source [N factor, comprising NH_4_^+^ or Trypticase (Try) levels]. Nitrogen source was included as a factor in the experimental arrangement of treatments to allow distinguishing between the net de novo synthesis of microbial AA from NH_4_^+^ incorporation (NH_4_^+^ treatment) and the sum of de novo synthesis of AA plus the uptake of preformed AA supplied by Try to the medium (Try treatment).

Approximately 0.5 L of rumen contents were sampled from the center of the rumen of each of two ruminally-cannulated non-pregnant, non-lactating, Holstein cows. The cows were fed ryegrass (*Lolium multiflorum*) hay [5.8% crude protein, 59.4% neutral detergent fiber (NDF), 6.0% total ashes, dry matter (DM) basis] once per day in the morning. Rumen contents were sampled before feeding around 10 am, and were filtered through two layers of synthetic cloth. The fluid fraction from both cows was pooled, and the pooled fluid, as well as the solid rumen content fractions from both cows, were transported to the laboratory in separate insulated containers.

The entire preparation of rumen inoculum was conducted under O_2_-free CO_2_ as previously described [16]. First, 100 mL of pooled rumen fluid were mixed with approximately 25 mL of rumen solids from each cow. The resulting mixture was discontinuously subjected to an eggbeater for 1 min (3 s bursts followed by 2 s breaks) to detach microorganisms adhered to solid particles. The blended mixture of fluid and solids was subsequently filtered through four layers of synthetic cloth to obtain the rumen inoculum. Four hundred microliters of rumen inoculum were delivered into each of 24 1-L serum bottles containing 400 mL of medium based on the Goering and van Soest [17] medium with the following additions: (i) 0.5 g/L yeast extract (Sigma-Aldrich 70161, India); (ii) 10 mL/L Pffenigs mineral solution [18]; (iii) 10 mL/L of VFA solution [19]. In addition, in 12 bottles (NH_4_^+^ treatment), Try in the Goering and van Soest medium [17] was replaced on an isonitrogenous basis by ammonium bicarbonate (Sigma-Aldrich 09830, Steinheim, Germany). In the remaining 12 bottles (Try treatment), Try (Becton, Dickinson and Company, Sparks, MD, US; 8.92% N on a dry matter basis) replaced ammonium bicarbonate in the Goering and van Soest medium [17] on an isonitrogenous basis. The extra bicarbonate anion added to the NH_4_^+^ medium as ammonium bicarbonate was compensated in the Try medium by adding an equimolar amount of sodium bicarbonate (4.25 g/L). The media composition is summarized in Table S1. Initial samples were taken and stored frozen at −20 °C for subsequent analysis of initial VFA concentration.

Six of the 12 bottles with each media contained 3.5 g of cellulose (Sigma C6288, Steinheim, Germany), and the other six bottles contained 3.5 g of starch (United States Biological, Swampscott, MA, US). The exact mass of each substrate was recorded. Finally, three bottles of each combination of medium and substrate contained no additive (Control treatment, AQ-) or 5 mg of AQ (AQ treatment, AQ+; Sigma-Aldrich A90004, Czech Republic).

Bottles were immediately sealed under O_2_-free CO_2_ after inoculation and placed in a shaking water bath at 39 °C and 60 cycles/min. The initial gas pressure above ambient was determined using a pressure transducer (Sper Scientific 840065, Scottsdale, AZ, USA) after allowing the bottles to warm up for 10 min. Bottles containing cellulose were incubated for 96 h and bottles containing starch were incubated for 72 h. Gas pressure was determined at 24, 48, 72 (all bottles), and 96 (cellulose bottles only) h of incubation.

### 2.2. Samples Processing

At 72 (starch bottles) or 96 (cellulose bottles) h of incubation, 20-mL gas samples were taken with a glass syringe and kept in vacutainers previously flushed with dinitrogen (N_2_) and evacuated to 0.2 atm, for subsequent analysis of gas composition. Bottles were then opened and final pH (Oakton^®^ pH 700 m, Singapore) and reducing potential (E*h*) (Schott Instruments BlueLine 31 Rx Ag/AgCl redox electrode) were immediately measured, and 1.5 mL of a 20% (*m*/*v*) sodium azide solution were added to arrest microbial activity. One milliliter aliquots were transferred to eppendorfs containing 0.2 mL of 20% (*m*/*v*) m-phosphoric acid (Merck 1.00546.0500, Darmstadt, Germany) or 1% (*v*/*v*) sulfuric acid, and stored at −20 °C until analyzed for VFA and NH_4_^+^ concentration, respectively.

Bottle contents were then emptied into pre-weighted centrifuge bottles and centrifuged at 10,956× *g* and 4 °C for 20 min to isolate the undigested solid residues. The supernatant was discarded, the pellets were lyophilized, and the centrifuge bottles weighted. The pellets dry mass was obtained by difference and the pellets were homogenized with mortar and pestle. Subsamples of approximately 50 mg were taken and stored at −20 °C for subsequent analysis of AA content.

### 2.3. Analytical Procedures

All analytical procedures were conducted in duplicate. All pellets were analyzed for DM [20]. Pellets from bottles in which cellulose was incubated were also analyzed for total ashes, total N [20] and NDF content [17]. The amount of pellet obtained from bottles incubated with starch was insufficient to analyze for total ashes, total N and starch content.

Gas composition and VFA concentration were analyzed as previously described [16]. Methane and H_2_ content in gas samples were analyzed in a Clarus 580 PerkinElmer GC equipped with a 60/80 Carboxen 1000 (Supelco, Bellefonte, PA, USA) packed column and a thermal conductivity detector. The carrier gas was N_2_ at 30 mL/min at an isothermal oven temperature of 180 °C. Samples for VFA analysis were thawed, vortexed, and centrifuged at 16,000 × *g* for 10 min. The supernatant was filtered through 0.45 µm pore cellulose filters into 2-mL GC vials, and 1 µL VFA samples were injected in a Clarus 580 PerkinElmer GC equipped with an Elite-FFAP (PerkinElmer, Shelton, CT, USA) capillary column and a flame ionization detector. Helium at 1.5 mL/min was the carrier gas. Initial temperature was 90 °C with a 12 °C/min ramp until 150 °C, which was held for 5 min. 2- and 3-methylbutyrate co-eluted and their concentration is reported as their sum.

Ammonium concentration was determined colorimetrically [21]. For analysis of AA, lyophilized pellet subsamples of approximately 20 mg were transferred to hydrolysis tubes and added 1 mL of a 6 M hydrochloric acid and 1% (*m*/*v*) phenol solution. The tubes were then gassed with N_2_, closed air-tightly, and incubated at 110 °C for 24 h [22]. Tube contents were then filtered through 0.45 µm cellulose filters and 20 µL of the filtrate diluted with ultrapure water to 500 µL. Ten microliters of the resulting dilution were derivatized with 6-aminoquinolyl-N-hydroxysuccinimidylcarbamate at 55 °C for 10 min using the Waters^®^ AccQ•Tag^TM^ Amino Acid Analysis Method (Waters Corporation, Milford, MA, US). Subsequently, 20 µL of derivatized sample were injected into a Hitachi L-7100 HPLC equipped with a C18 Sunshell (Biotech, Onsala, Sweden) column and a UV-Visible detector operating at 254 nm. Oven temperature was 36 °C with sodium acetate at a pH of 6.3 and 60% (*v*/*v*) acetonitrile as mobile phases. A standard AA mix containing all AA except Gln, Asn and Trp at 100 µM concentration (Waters Corporation, Milford, MA, US) was used to fit standard curves for each AA [16].

### 2.4. Calculations

Total gas production as number of moles at each time point was calculated using the ideal gas law [23], with total gas pressure calculated as the measured gas pressure minus the initial gas pressure determined after 10 min of incubation, and a 600-mL headspace volume and 312 K. Final production of CH_4_ and accumulation of H_2_ were calculated by multiplying the number of moles of total gas produced by its concentration. Concentration of CH_4_ and H_2_ in total gas were expressed discounting the initial number of moles of CO_2_ in the headspace calculated as explained above. The E*h* recorded at the end of the incubations was corrected to the Standard Hydrogen Electrode (SHE) by adding 197 mV [24]. Net total and individual VFA production was calculated by subtracting initial from final VFA concentration.

Apparent dry matter (DM) digestibility was calculated from the initial DM amount of each substrate and the amount of undigested residue DM. True digestibility of organic matter (OM) and net microbial biomass production were calculated for the cellulose-containing incubations as described before [16], as follows. Incubated cellulose contained 95% DM and 97.7% NDF (DM basis), and undetectable total ashes. The amount of undigested cellulose substrate on a DM basis was calculated by dividing the NDF content in the undigested incubation residue by cellulose NDF content expressed as a proportion. As incubated cellulose had undetectable total ash content, undigested cellulose DM was assumed to be equal to undigested cellulose OM:

(1) Undigested cellulose (mg DM)=undigested cellulose (mg OM)=undigested pellet (mg DM)×(NDF% in undigested pellet÷100)÷0.977

For the above calculation, it was assumed that undigested cellulose had the same composition that the cellulose substrate incubated. True OM digestibility was calculated by dividing the difference between OM incubated in cellulose and undigested cellulose OM calculated as by Equation (1), and expressing the result as a percentage:

(2) OM true digestibility=[incubated cellulose (mg OM)−undigested cellulose (mg OM)]×100÷incubated cellulose (mg OM)

Net production of microbial DM was calculated by subtracting the mass of undigested cellulose DM from the total mass of the undigested solid residue DM:

(3) Microbial biomass (mg DM)=undigested solid residue (mg DM)−undigested cellulose (mg DM)

True OM digestibility and net production of microbial DM could not be calculated for starch incubations as the amount of indigested residue was insufficient to conduct an analysis of starch content.

Net production of microbial N and AA were calculated by assuming that all N and AA present in the undigested residue resulted from microbial growth, as the initial content of N and AA in the substrate and the inoculum was minimized by using pure cellulose or starch as N-devoid substrates, and by reducing the amount of inoculum from 20% (*v*/*v*) [17] to 0.1% (*v*/*v*). In the NH_4_^+^ treatments, the total content of microbial AA in the solid residue was considered to be equal to the net de novo synthesis of microbial AA, as no preformed AA were present in the medium. In the Try treatments, the net production of microbial AA was considered to be equal to the sum of the incorporation of preformed AA from Try plus net de novo synthesis of AA from NH_4_^+^ resulting from the deamination of AA in Try and recycled microbial protein [9,10,25].

Flows and sinks of [H] were quantified as the balance of pairs of reducing equivalents ([2H]) produced (*[2H]_produced_*) and incorporated (*[2H]_incorporated_*) in the catabolism of glucose (as the product of cellulose and starch hydrolysis) to VFA and gases (Table S2) [16]. Formate and heptanoate were not considered because they were not determined, and caproate was not considered as there are two different pathways of caproate formation with different implications to the electron balance [26] and the proportion of caproate formed in each pathway was not determined. Recovery of [2H] (*[2H]_recovery_*) was calculated as the ratio of total *[2H]_incorporated_* to *[2H]_produced_* expressed as a percentage [16].

For the incubations with NH_4_^+^ as N source, the production and incorporation of [2H] was also calculated for the synthesis of AA from glucose, CO_2_ (generated from bicarbonate in the medium), VFA added to the medium, ATP carbon (in the case of His), and NH_4_^+^ (Table S3) [16]. Calculation of production and incorporation of [2H] in the synthesis of each AA was conducted based on reported biosynthetic pathways [12,13,27,28,29]. The existence of more than one biosynthetic pathway for some AA has implications with regards to the production and incorporation of [2H] [5,13]. Various biosynthetic pathways were therefore considered for some AA. In other cases, only the main pathway based on previous results with rumen microorganisms was considered (Table S3). Transaminations were considered to indirectly incorporate 1 mol of [2H] per mol of NH_3_ because amination in the synthesis of the AA donating NH_3_ in the transamination reaction, generally Glu or Ala, would incorporate 1 mol of [2H] [13]. Maximal and minimal net [2H] incorporation into the synthesis of each individual AA were considered for calculating the maximal and minimal net [2H] incorporation into the synthesis of total AA. Overall maximal and minimal production, incorporation and recovery of [2H] was calculated considering [2H] produced and incorporated into VFA and gases, plus maximum or minimum [2H] produced and incorporated into total AA synthesis [16]. Partial recoveries into each [2H] sink considering maximal recovery of [2H] into AA synthesis were also calculated.

### 2.5. Statistical Analyses

There were three replicates per treatment per incubation and three incubation runs conducted on different days. Responses were modeled with a mixed linear model including the three treatments as fixed main effects, their double and triple interactions, and the random effect of the incubation run:

(4) Y=μ+M+E+N+M×E+M×N+E×N+M×E×N+I+ε

where *Y* is a response variable, *M* is the methanogenesis inhibition fixed effect (AQ- or AQ+), *E* is the carbon and energy substrate fixed effect (cellulose or starch), *N* is the *N* source fixed effect (NH_4_^+^ or Try), *M* × *E*, *M* × *N* and *E* × *N* are the double interactions fixed effects, *M* x *E* × *N* is the triple interaction fixed effect, *I* is the random effect of the incubation run, and *ε* is the random error.

Production and incorporation of [2H] in de novo synthesis of AA was analyzed for the NH_4_^+^ treatments only, as in the Try treatments an unknown part of microbial AA originated from direct incorporation of preformed AA from the medium. Therefore, a reduced model with two main effects and their interactions was used for [2H] in AA synthesis:

(5) [2H]produced or incorporated AA=μ+M+E+M×E+I+ε

With variables defined as in Equation (4).

Total gas pressure was modeled as a function of time including the random effect of the incubation bottle as the repeated measures variable:

(6) Y=μ+M+E+N+t+M×E+M×N+E×N+M×t+E×t+N×t+M×E×N+M×E×t+M×N×t+E×N×t+M×E×N×t+I+B+ε

where *t* is the fixed effect of time, *M* × *t*, *E* × *t* and *N* × *t* are the double interactions between methanogenesis inhibition, carbon and energy source and N source with time, respectively, *M* × *E* × *N*, *M* × *E* × *t*, *M* × *N* × *t*, *E* × *N* × *t* are the triple interactions, *M* × *E* × *N* × *t* is the quadruple interaction, and *B* is the random effect of the incubation bottle, with the rest of the effects defined as in Equation (4).

Significance was declared at *p* < 0.05 and tendencies at 0.05 ≤ *p* < 0.10. Non-significant interactions (*p* > 0.10) were removed and the reduced models re-fitted. If higher order interaction were maintained in the model, lower order interactions were also kept in the model (hierarchical principle [30]). If interactions were significant (*p* < 0.05) or tended to significance (0.05 ≤ *p* < 0.10), LSD multiple comparisons are provided to facilitate their understanding.

Total *[2H]_recovery_* was regressed against the linear and quadratic effect of CH_4_ production, the type of energy substrate, and their interaction:

(7) $$\left[2H\right]recovery=\mu +CH4+CH4^{2}+E+E\times CH4+I+\varepsilon$$

where *[2H]_recovery_* is equal to *[2H]_incorporated_* expressed as a percentage of total *[2H]_produced_*; CH_4_ is CH_4_ production in mmol; and *µ*, *E*, *I,* and *ε* are defined as in Equation (4). For this regression, the maximum *[2H]_recovery_* into AA synthesis was considered. If quadratic and interaction terms were non-significant (*p* ≥ 0.10), they were removed and the reduced model refitted.

JMP^®^ 13.2.1 [31] was used for all statistical analyses.

## 3. Results

### 3.1. Digestion and Fermentation

9, 10-Anthraquinone decreased apparent DM digestibility with the cellulose (*p* < 0.001) but not with the starch substrate (interaction *p* < 0.001), and decreased true OM digestibility (which was determined with the cellulose substrate only; *p* < 0.001; Table 1). There was an interaction (*p* < 0.001) between methanogenesis inhibition and the energy substrate on total gas pressure, with greater total gas pressure evolving in the Control treatments in the cultures growing on cellulose (*p* < 0.001), and conversely greater total gas pressure evolving with AQ in the cultures growing on starch (*p* < 0.001; Table 1 and Figure S1). 9, 10-Anthraquinone decreased CH_4_ production (*p* < 0.001; Table 1). In starch incubations, AQ had a greater effect on H_2_ accumulation with Try than with NH_4_^+^, whilst the effect of AQ on H_2_ accumulation was similar with both N sources in cellulose incubations (triple interaction *p* < 0.001).

Starch decreased final pH (*p* < 0.001), especially with AQ (interaction *p* < 0.001; Table 1). Final E*h* was decreased by AQ (*p* < 0.001), especially with starch (interaction *p* < 0.001). Cellulose (*p* < 0.001), NH_4_^+^ (*p* < 0.001) and AQ (*p* < 0.05) decreased total VFA production. 9, 10-Anthraquinone increased acetate molar percentage with cellulose and NH_4_^+^ (*p* < 0.05) but decreased it with starch and Try (*p* < 0.05; triple interaction *p* < 0.01). Propionate molar percentage was greater with starch (*p* < 0.001) and was decreased by AQ (*p* < 0.001). 9, 10-Anthraquinone increased butyrate molar percentage (*p* < 0.001), especially with starch (interaction *p* < 0.001). 9, 10-Anthraquinone increased isobutyrate molar percentage with cellulose (*p* < 0.05) but not with starch (interaction *p* < 0.001). Cellulose, NH_4_^+^ and AQ all increased 2- plus 3-methylbutyrate molar percentage (*p* < 0.001). 9, 10-Anthraquinone increased valerate molar percentage with cellulose (*p* < 0.05) but decreased it with starch and Try (*p* < 0.05; triple interaction *p* < 0.001). 9, 10-Anthraquinone increased the acetate to propionate molar ratio (*p* < 0.001), with the increase being greater with cellulose than with starch (interaction *p* < 0.001). 9, 10-Anthraquinone increased NH_4_^+^ concentration with cellulose (*p* < 0.05) and decreased it with starch (*p* < 0.05; interaction *p* < 0.001).

### 3.2. Microbial Biomass Production and Amino Acidic Composition

9, 19-Anthraquinone decreased microbial N production in cellulose cultures (*p* < 0.001; Table 2). 9, 10-Anthraquinone increased total microbial AA-N with starch and NH_4_^+^ only (*p* < 0.05; triple interaction *p* = 0.083). With cellulose and NH_4_^+^, AQ increased the percentage of Asp in total microbial AA (*p* < 0.05; triple interaction *p* = 0.017) and decreased Arg (*p* < 0.05; triple interaction *p* = 0.098). 9, 10-Anthraquinone increased Ser (*p* < 0.001) and Gly (*p* < 0.001), and decreased Pro (*p* < 0.05) and Leu (*p* < 0.05) percentages in total microbial AA with cellulose only (interaction *p* < 0.05). 9, 10-Anthraquinone increased Ala (*p* = 0.014), and decreased His (*p* = 0.036), Tyr (*p* < 0.001) and Phe (*p* = 0.006), and tended to decrease Lys (*p* = 0.093) percentages in total AA, with all energy and N sources.

**Figure 1:**
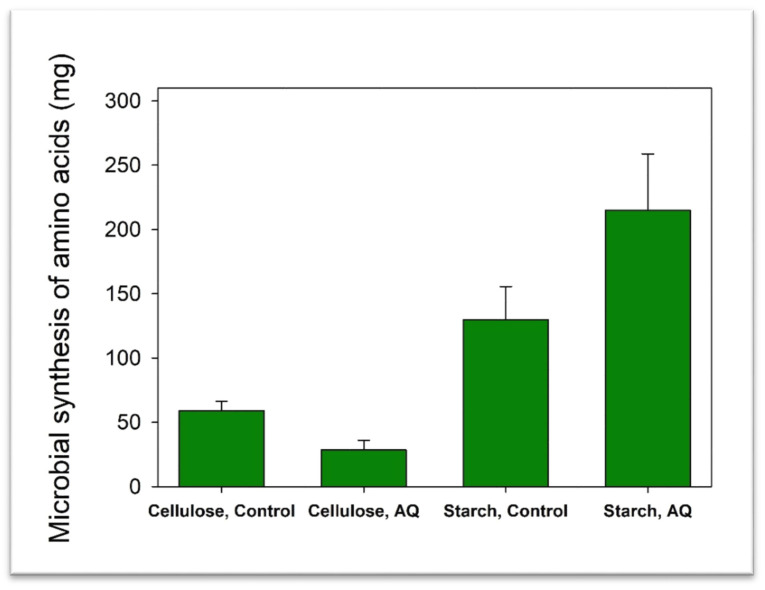
Net de novo synthesis of microbial amino acids in mixed rumen batch cultures growing on cellulose or starch with functional methanogenesis (Control) or with methanogenesis inhibited by 9, 10-anthraquinone (AQ), depicting an interaction (*p* < 0.05) between methanogenesis inhibition and the energy and carbon substrate.

No preformed AA were available in the medium in the NH_4_^+^ treatments, thus it was assumed that all microbial AA were synthesized de novo. With cellulose, AQ consistently decreased de novo synthesis of all AA, although this was not statistically significant (*p* > 0.05; Table 3). Conversely, with starch, AQ increased (*p* < 0.05) de novo synthesis of Asp, Glu, Ser, Gly, Arg, Thr, Ala, Pro, Val, Ile, Leu, Lys and Phe, and total AA (Figure 1), and numerically increased the synthesis of His and Tyr (*p* > 0.05).

In the Try treatments, microbial AA resulted from both de novo synthesis and incorporation of preformed AA from the medium. Both with cellulose and with starch, AQ numerically (*p* > 0.05) but consistently decreased the sum of de novo synthesis plus incorporation of all AA.

### 3.3. Metabolic Hydrogen Balance

9, 10-Anthraquinone decreased the production of [2H] associated to acetate production with cellulose (*p* < 0.05) but not with starch (*p* < 0.05; interaction *p* = 0.068; Table 4), and decreased the production of [2H] associated to propionate production with all energy and N sources (*p* < 0.001). 9, 10-Anthraquinone decreased the production of [2H] associated to butyrate production numerically (*p* > 0.05) with the cellulose substrate, and increased it with the starch substrate (*p* < 0.05; interaction *p* < 0.01). The production of [2H] associated to valerate production was decreased (*p* < 0.05) by AQ with the starch substrate only, and especially with Try (triple interaction *p* < 0.001). Total production of [2H] from VFA production was numerically decreased (*p* > 0.05) by AQ with cellulose and numerically increased (*p* > 0.05) with starch (interaction *p* = 0.021).

9, 10-Anthraquinone decreased the incorporation of [2H] into propionate with all energy and N substrates (*p* < 0.001; Table 4). Incorporation of [2H] into butyrate formation was numerically decreased by AQ with cellulose (*p* > 0.05) and increased with starch (*p* < 0.05; interaction *p* < 0.01). 9, 10-Anthraquinone did not affect the incorporation of [2H] into valerate formation with cellulose and decreased it with starch (*p* < 0.05), especially with Try (triple interaction *p* < 0.05). 9, 10-Anthraquinone decreased [2H] incorporation into CH_4_ formation with starch (*p* < 0.05) and with cellulose and Try (*p* < 0.05), but only numerically with cellulose and NH_4_^+^ (*p* > 0.05). Incorporation of [2H] into H_2_ was increased by AQ with starch (*p* < 0.05), especially with Try (triple interaction *p* < 0.001). Total [2H] incorporation into VFA and gases was numerically decreased by AQ with cellulose (*p* > 0.05) and unaffected with starch (*p* > 0.05). With all sources of energy and N, AQ decreased the recovery of [2H] in VFA and gases (*p* < 0.001).

**Figure 2:**
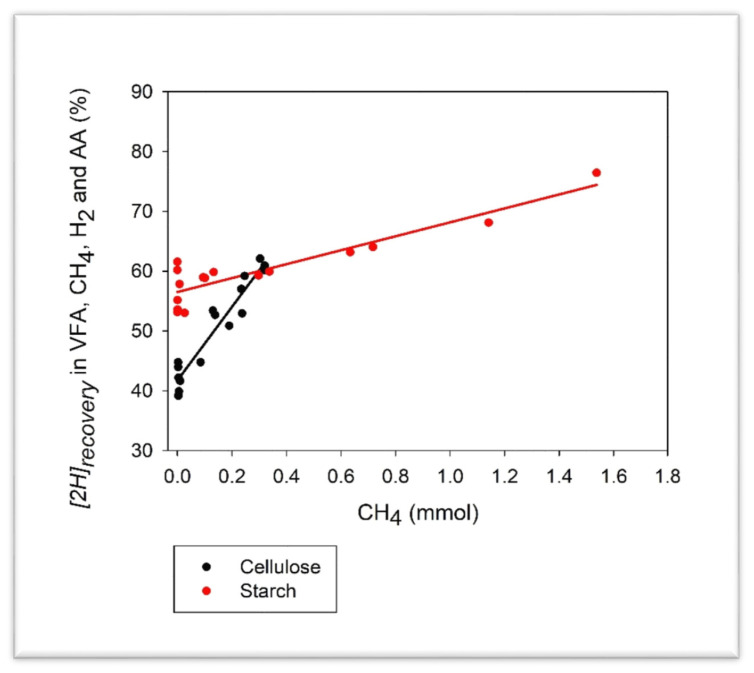
Response in the recovery of metabolic hydrogen in volatile fatty acids (VFA), methane (CH_4_), dihydrogen (H_2_), and amino acids (AA) to the inhibition of methanogenesis by 9, 10-anthraquinone in mixed rumen batch cultures growing on cellulose or starch: *y* = 48.9 (±0.60; *p* < 0.001) – 1.39 (±0.50; *p* = 0.009) C + 36.8 (±2.56; *p* < 0.001) CH_4_ + 24.7 (±2.56; *p* < 0.001) C × (CH_4_ – 0.234).

9, 10-Anthraquinone decreased the minimum net incorporation of [2H] into AA synthesis with starch only (*p* < 0.05; interaction *p* < 0.01; Table 4). Conversely, the maximum net incorporation of [2H] into AA synthesis was increased by AQ with starch only (*p* < 0.05; interaction *p* < 0.01). The recovery of [2H] into VFA, gases and AA (minimum or maximum) was decreased by AQ (*p* < 0.001), especially with cellulose (interaction *p* < 0.05). The decrease in the recovery of [2H] into VFA, gases and AA as a response to the decrease in CH_4_ production was greater for cellulose than for starch (interaction *p* ≤ 0.01; Figure 2).

**Figure 3:**
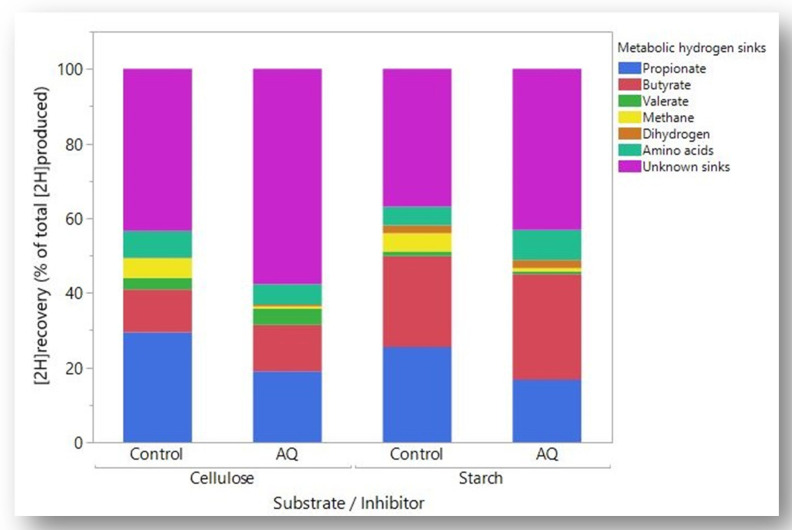
Partial recoveries of metabolic hydrogen (ratio of metabolic hydrogen incorporated into each sink to total metabolic hydrogen produced, expressed as percentage) in mixed rumen batch cultures growing on cellulose or starch with functional methanogenesis (Control) or with methanogenesis inhibited by 9, 10-Anthraquinone (AQ).

A summary of partial [2H] recoveries by treatment, calculated with the maximum recovery of [2H] in AA synthesis, is shown in Figure 3. 9, 10-Anthraquinone decreased the partial recovery of [2H] in propionate (*p* < 0.001) and CH_4_ (*p* < 0.001), and increased the partial recovery of [2H] in butyrate (*p* < 0.001), especially with starch (interaction *p* < 0.001). 9, 10-Anthraquinone increased the partial recovery of [2H] in valerate with cellulose (*p* < 0.001) but decreased it with starch (*p* < 0.05; interaction *p* < 0.001). The partial recovery of [2H] in H_2_ was unaffected by AQ (*p* = 0.14). The partial recovery of [2H] in AA was unaffected by AQ with cellulose (*p* = 0.30) and tended (*p* < 0.060) to increase with starch (interaction *p* = 0.039). As also reflected by Figure 2, AQ increased the recovery of [2H] in unknown sinks (*p* < 0.001), especially with cellulose (interaction *p* = 0.013).

## 4. Discussion

The central hypothesis of this study related to the redirection of [H] toward AA synthesis when methanogenesis is inhibited in rumen fermentation. Inhibiting methanogenesis typically enhances propionate as an alternative [H] sink [8,32]. In previous experimental work, AQ had not promoted the redirection of [H] from CH_4_ to propionate formation [16], and it was therefore thought that it could promote other [H] sinks such as AA if enough energy and carbon were available. The effects of AQ on fermentation seem to be concentration and system-dependent, with doses of up to 5 ppm increasing propionate molar percentage in batch cultures [33], and 10 ppm decreasing it in batch [16] and continuous [33] cultures. In a semi-continuous culture study, AQ added daily to an initial concentration of 13.7 ppm did not affect propionate molar percentage [26]. In the present study, AQ at 12.5 ppm inhibited propionate as an alternative [H] sink to CH_4_.

We used pure cellulose and starch as N-devoid energy substrates and decreased the amount of inoculum to only 0.1% (*v*/*v*), to allow quantifying microbial N as all of the N present in the solid residue after the incubations [16]. Including NH_4_^+^ or Try as a factor in the experimental arrangement of treatments allowed distinguishing between the net de novo synthesis of microbial AA (NH_4_^+^ treatment) and the sum of de novo synthesis of AA plus the uptake of preformed AA from the medium (Try treatment). It has been shown that the inclusion of AA and peptides in the medium decreases the proportion of AA synthesized de novo from NH_4_^+^ [34,35,36].

In rumen batch cultures, CH_4_ is typically the main sink of [H] if methanogenesis is not inhibited [8]. In the present study, the importance of CH_4_ as [H] sink was surpassed by propionate and butyrate in the Control treatments both with the cellulose and the starch substrates (Figure 3). This coincides with a previous study using similar experimental conditions, in which it had been speculated that the small amount of inoculum or the chemically pure nature of the substrates might have affected the growth of methanogens [16]. In the present study, butyrate was an important [H] sink, especially with starch, and the response of butyrate to AQ in the cultures growing on starch was greater than in the cultures growing on cellulose. Similarly, García-López et al. [33] reported an increase in the response of butyrate molar proportion to AQ in rumen batch cultures as the proportion of concentrate in the substrate increased. An unexpected result of the present research was that when methanogenesis was inhibited, the decrease in *[2H]_recovery_* with starch was less severe than with cellulose, which was mainly due to the greater response in butyrate with starch, and also of AA if they acted as [H] sinks, i.e., the net incorporation of [2H] in AA biosynthesis was closer to the theoretical maximum than to the minimum. Implications of greater rumen butyrate production in response to methanogenesis inhibition include potential increased milk fat synthesis in dairy cows [37,38,39], although milk fat yield was decreased by butyrate infusion in a recent study [40].

In previous work, it had been hypothesized that inhibiting methanogenesis would stimulate the incorporation of [H] into AA biosynthesis, but that could not be demonstrated using pure cellulose as substrate [16]. The question posed for the present research was if AA biosynthesis could become limited by energy and/or carbon when the inhibition of CH_4_ formation enhanced the availability of [H]. In the present study, it was shown that in cultures growing on starch, but not on cellulose, inhibiting methanogenesis with AQ stimulated de novo synthesis of all microbial AA except for His and Tyr. 9, 10-Anthraquinone increased de novo synthesis of total AA by 65% with starch, but numerically decreased it by 52% with cellulose. In a previous study, inhibiting methanogenesis with chloroform resulted in an increase in the concentration of almost all AA in the fluid phase of rumen contents of steers fed a hay diet, and only some AA with a mixed diet [41]. The authors of that study suggested that the increases in the concentration of AA in the fluid phase, along with increases in the molar percentages of isoacids, indicated increases in proteolysis of dietary protein with likely greater fermentation of AA.

Whether or not the increase in AA synthesis with AQ in the starch cultures also increased the incorporation of [2H] into AA synthesis depends on the predominant biosynthetic pathways. If all AA were to be synthesized through pathways that result in a net release of [2H], then the increase in AA synthesis by AQ would obviously result in an increase in [2H] release in starch cultures. If, on the contrary, AA synthesis incorporated the theoretically possible maximum [2H], the net [2H] incorporated into AA synthesis in starch cultures would have been increased by AQ. Although in the present experiment the biosynthetic pathways of the different AA were not determined, it is unlikely that an increase in [H] availability caused by inhibiting methanogenesis, as evidenced by increased H_2_ accumulation and decreased E*h*, could stimulate [H]-releasing pathways [32]. Thus, it is thought that the [2H] balance of AA synthesis was likely closer to the maximum net incorporation of [H] into AA synthesis than to the minimum. The fact that nearly all AA responded similarly to AQ in the starch cultures, as the AA profile was little affected, also supports a general mechanism of stimulation of AA synthesis related to increased availability of [H].

Microbial protein recycling in mixed rumen batch cultures has been shown to occur extensively [25], implying the simultaneous occurrence of amination and deamination. An increase in de novo synthesis of AA by AQ in the starch cultures could have been mediated by an inhibition of deamination, a stimulation of amination, or a combination of both. Previous research has shown that the extent of the effects of chloroform, carbon monoxide, and ionophores on deamination, and the mechanisms involved, differ [9]. Both chloroform and carbon monoxide were potent inhibitors of CH_4_ formation in mixed rumen batch cultures fermenting Try, but carbon monoxide, a hydrogenase inhibitor, was particularly inhibitory to the deamination of branched-chain AA. Chloroform and the ionophores monensin and lasalocid also inhibited deamination but were not selective against branched-chain AA [9]. The inhibitory effects of carbon monoxide on deamination of branched-chain AA were confirmed in another study [10]. Hino and Russell [11] showed that the inhibition of the deamination of branched-chain AA by carbon monoxide was mediated by an increase in the NADH/NAD^+^ ratio.

In the starch cultures of our study, the profile of AA synthesized de novo and the molar percentages of branched-chain VFA and valerate, which are precursors of branched-chain AA Leu, Ile and Val [42,43,44], were little affected by AQ, therefore, AQ stimulated amination or inhibited deamination non-selectively. The fact that cultures growing on starch, but not on cellulose, responded to AQ with greater net synthesis of AA, points towards a general stimulation of amination allowed by greater availability of [H] resulting from methanogenesis inhibition along with a simultaneously augmented supply of energy and carbon from the more fermentable substrate, rather than to an inhibition of deamination.

In contrast to cultures growing on starch, AQ affected the profile of AA synthesized de novo in cultures growing on cellulose, with increases in the percentages of Asp, Ala, Ser, and Gly in total AA, and decreases in Arg, Pro, Leu, Tyr, and Phe. Aspartate can be produced by transamination of Glu to oxaloacetate [27,28], with oxaloacetate being an intermediate of the randomizing pathway of propionate production [45]. 9, 10-Anthraquinone actually decreased propionate molar percentage and production both with cellulose and starch, however, it is unknown how AQ affected the partition of carbon flowing through the randomizing and non-randomizing pathways. Alanine is synthesized by transamination of pyruvate with Glu or Val [28]. Pyruvate can be obtained from glycolysis [12] but can also be formed from the reductive carboxylation of acetate [27,29,46]. It is possible that greater availability of [H] with AQ could have stimulated acetate reductive carboxylation to pyruvate. Glycine is synthesized from Ser [27,28], which may explain the simultaneous increase in the percentages of Ser and Gly in total AA with AQ in cultures growing on cellulose.

Leucine can be derived from pyruvate [27,28], but also from the direct carboxylation of 3-methylbutyrate (isovalerate) followed by amination [29,42,47]. In agreement with the decrease in the percentage of Leu in total de novo synthesized AA in cellulose grown cultures, the molar percentage of 2- plus 3-methylbutyrate was increased by AQ, likely indicating a decrease in the amination of 3-methylbutyrate for its incorporation into Leu synthesis. In rumen fermentation, 2-methylbutyrate is a precursor of Ile [44], and isobutyrate is a precursor of Val [43]. The same as with Leu, AQ decreased the percentages of Ile and Val in total de novo synthesized AA, although non-significantly, and increased the molar percentages of their precursors isobutyrate and 2-methylbutyrate. This suggests a general effect of AQ decreasing the amination and incorporation of branched-chain VFA into de novo synthesis of branched-chain AA in the cultures growing on cellulose.

Tyrosine and Phe biosynthesis share the shikimate pathway in many organisms [28] and in rumen cultures [27], but Phe can also be synthesized from phenylacetate and phenylpyruvate [48,49], and Tyr produced from Phe [50]. The simultaneous decrease in the percentage of Tyr and Phe caused by AQ in the cultures grown on cellulose agrees with the common synthetic pathways for these AA in rumen fermentation.

The percentage of Arg and Pro in total de novo synthesized AA was decreased by AQ in cultures growing on cellulose. Both Arg and Pro derive most of their carbon from Glu [28,29], although the percentage of Glu in total de novo synthesized AA was unaffected by AQ in cultures growing on cellulose. The conversion of Glu to Arg and Pro requires ATP [28]. The decrease in VFA production caused by AQ likely decreased ATP generation, although this would be thought to also affect the synthesis of other AA.

Depending on biosynthetic pathways, the potential importance of AA as a [H] sink can be minor but not negligible, and it tended to increase with starch as energy and carbon substrate. Inhibiting methanogenesis in vitro has been shown to consistently decrease the recovery of [2H] into VFA and gases [8], and AA synthesis could explain some of the unaccounted [2H] sinks in high concentrate diets rich in starch. The present research with chemically pure substrates can be considered a proof of concept study, and the stimulation of AA synthesis as a consequence of inhibiting methanogenesis in highly fermentable diets would need to be confirmed in future experiments using real ruminant diets as substrates and rumen inoculum adapted to concentrates, as well as other inhibitors of methanogenesis.

If the increase observed in the synthesis of AA with AQ in the present study is a general consequence of inhibiting methanogenesis with highly fermentable diets, this could allow partially replacing expensive plant protein supplements with non-protein N sources such as urea. Generally, however, high-concentrate diets used in feedlots are relatively low in protein, and animal protein requirements can be met without extensive use of plant protein supplements. Mixed diets fed to dairy cows are higher in protein, with a current recommendation of urea not replacing more than 20% of dietary crude protein [51]. A larger replacement of plant protein supplements with urea may be a greater opportunity with those diets when simultaneously inhibiting methanogenesis, compared to lower protein diets. Therefore, it would be important to evaluate if inhibiting methanogenesis could also enhance microbial synthesis of AA with mixed diets used for feeding dairy cows.

Ruminants have the nutritional flexibility to utilize non-protein N. However, the production of excess NH_4_^+^ in the rumen from degradation of dietary protein and subsequent fermentation of AA can exceed the capacity of rumen microbes to incorporate it into microbial protein synthesis. Excess NH_4_^+^ is absorbed both as NH_4_^+^ and NH_3_, converted to urea by the liver, and partly excreted in urine [15], causing atmospheric and water pollution [52]. If inhibiting rumen methanogenesis could increase the incorporation of NH_4_^+^ into microbial AA with some diets, there could potentially be a dual benefit in simultaneously decreasing both CH_4_ and N emissions.

## 5. Conclusions and Implications

Inhibiting CH_4_ production in the rumen may increase the synthesis of microbial AA with the most fermentable ruminant diets, which could benefit both ruminant production and the environment by decreasing energy losses as CH_4_ and the needs of plant protein supplements, and the release of CH_4_ and N to the environment. Future research should evaluate the interaction between methanogenesis inhibition and microbial synthesis of AA with a broader range of diets used in ruminant production, different types of animals, as well as other inhibitors of rumen methanogenesis.

## Figures and Tables

**Table 1:** Effects of the addition of 9, 10-anthraquinone to rumen batch cultures incubated with cellulose or starch as carbon and energy sources and ammonium or trypticase as nitrogen sources on digestion and fermentation variables.

Responses	Treatments								M	C	N	M × C	M × N	C × N	M × C × N	RMSE
	Cellulose				Starch											
	NH_4_^+1^		Try		NH_4_^+^		Try									
	AQ-	AQ+	AQ-	AQ+	AQ-	AQ+	AQ-	AQ+								
Apparent DM^2^ digestibility	18.4^b4^	6.74^c^	18.7^b^	3.82^c^	83.7^a^	84.9^a^	79.4^a^	82.9^a^	***^5^	***	***	***	NS	NS	NS	6.67
True OM digestibility (%)	22.7^a^	12.9^b^	25.6^a^	12.3^b^	ND	ND	ND	ND	***	-	NS	-	NS	-	-	7.43
Total gas (nmol g substrate^−1^ h^−1^)	260^cd^	129^e^	294^c^	197^de^	1460^b^	1521^b^	1477^b^	1638^a^	NS	***	**	***	+	NS	NS	74.8
CH_4_ (µmol)	236^cd^	105^d^	410^bc^	84.4^d^	573^b^	225^cd^	1005^a^	242^cd^	***	***	*	*	*	NS	NS	252
H_2_ (µmol)	19.7^e^	44.5^e^	23.2^e^	75.4^e^	1003^c^	1205^b^	800^d^	1611^a^	***	***	NS	***	***	NS	***	157
CH_4_ (mol/100 mol total gas produced)	3.39^b^	1.06^d^	4.76^a^	1.11^d^	2.28^c^	0.94^d^	3.96^b^	0.90^d^	***	**	***	*	***	NS	NS	0.75
H_2_ (mol/100 mol total gas produced)	0.29^f^	1.39^e^	0.35^f^	1.43^e^	3.73^e^	4.31^b^	2.98^d^	5.36^a^	***	***	NS	NS	***	NS	***	0.49
pH	6.79^c^	6.91^a^	6.84^bc^	6.89^ab^	6.43^e^	6.23^f^	6.56^d^	6.23^f^	***	***	*	***	**	NS	NS	0.067
E*h*	−170^bc^	−193^de^	−173^cd^	−189^cd^	−144^a^	−228^f^	−150^ab^	−210^ef^	***	NS	NS	***	NS	NS	NS	21.6
Total VFA (mM)	22.6	13.9	30.2	21.8	51.9	52.0	67.1	64.4	*	***	***	NS	NS	NS	NS	9.55
Acetate (molar%)	54.5^b^	60.0^a^	55.4^b^	56.4^b^	39.9^c^	38.7^cd^	39.0^c^	36.9^d^	+	***	**	***	**	NS	+	2.01
Propionate (molar%)	29.0^a^	19.5^d^	25.6^b^	19.6^d^	29.3^a^	21.8^c^	29.6^a^	22.3^c^	***	***	NS	NS	+	*	+	1.95
Butyrate (molar%)	11.3^ef^	13.3^d^	10.4^f^	12.3^de^	28.4^b^	36.8^a^	26.4^c^	35.6^a^	***	***	***	***	NS	NS	NS	1.37
Isobutyrate (molar%)	1.28^d^	1.59^c^	2.23^b^	2.69^a^	0.57^e^	0.51^e^	1.46^cd^	1.38^cd^	*	***	***	***	NS	NS	NS	0.27
2- and 3-methylbutyrate (molar%)^3^	1.64	2.69	2.79	3.94	0.94	1.40	2.17	3.10	***	***	***	NS	NS	NS	NS	0.28
Valerate (molar%)	1.50^d^	2.28^c^	3.23^b^	4.47^a^	0.76^e^	0.52^e^	1.27^d^	0.53^e^	***	***	***	***	NS	***	***	0.11
Caproate (molar%)	0.77^a^	0.63^a^	0.34^b^	0.61^a^	0.17^b^	0.16^b^	0.12^b^	0.16^b^	NS	***	NS	NS	+	NS	NS	0.27
Ac/Pr (mol/mol)	1.90^c^	3.09^a^	2.19^b^	2.90^a^	1.37^e^	1.79^cd^	1.33^e^	1.68^d^	***	***	NS	***	*	NS	+	0.22
NH_4_^+^ (mM)	11.7^b^	13.6^a^	9.15^c^	10.2^c^	5.87^d^	5.50^d^	3.42^e^	2.18^f^	NS	***	***	***	NS	NS	NS	1.19

^1^ NH_4_^+^, ammonium; Try, trypticase; AQ-, Control (non-inhibited methanogenesis) treatment; AQ+, 9, 10-Anthraquinone (methanogenesis inhibited) treatment; M, methanogenesis inhibition (control or 9, 10-anthraquinone); C, carbon source (cellulose or starch); N, nitrogen source (ammonium or trypticase); RMSE, root mean square of the error. ^2^ DM, dry matter; OM, organic matter; CH_4_, methane; H_2_, dihydrogen; E*h*, reducing potential; VFA, volatile fatty acids; Ac/Pr, acetate to propionate molar ratio. ^3^ Sum of 2- and 3-methylbutyrate as they co-eluted from the GC column. ^4^ Unlike superscripts within a row indicate significant (*p* < 0.05) differences when interactions (*p* < 0.10) are present. ^5^ NS, *p* > 0.10; +, 0.05 ≤ *p* < 0.10; *, 0.01 ≤ *p* < 0.05; **, 0.001 ≤ *p* < 0.01; ***, *p* < 0.001; ND, mean was not determined; -, effect was not determined.

**Table 2:** Effects of the addition of 9, 10-anthraquinone to rumen batch cultures incubated with cellulose or starch as carbon and energy sources and ammonium or trypticase as sources of nitrogen on microbial biomass production and amino acidic composition.

Responses	Treatments								M^1^	C	N	M × C	M × N	C × N	M × C × N	RMSE
	Cellulose				Starch											
	NH_4_^+^		Try		NH_4_^+^		Try									
	AQ-	AQ+	AQ-	AQ+	AQ-	AQ+	AQ-	AQ+								
Microbial DM^2^ (mg)	122	133	188	190	ND^3^	ND	ND	ND	NS	-	*	-	NS	-	-	84.3
Microbial N (mg)	8.28^b4^	4.53^d^	11.7^a^	6.17^c^	ND	ND	ND	ND	***	-	***	-	+	-	-	1.44
Total microbial AA-N (mg)	6.82^d^	3.37^d^	8.79^cd^	4.56^d^	14.6^bc^	24.7^a^	17.8^b^	15.0^bc^	NS	***	NS	*	+	NS	+	6.98
Amino acid (g/100 g total AA)																
Asp	14.9^b^	19.8^a^	13.6^bc^	13.2^bc^	13.1^bc^	11.8^c^	11.6^c^	12.0^bc^	NS	***	**	+	NS	*	*	2.94
Glu	14.7^ab^	14.6^ab^	13.9^abc^	15.7^a^	13.2^bc^	13.5^bc^	14.2^abc^	15.7^a^	*	**	NS	NS	+	NS	NS	1.93
Ser	5.48^b^	9.41^a^	5.82^b^	8.40^a^	5.17^b^	4.85^b^	5.04^b^	5.54^b^	***	***	NS	***	NS	NS	NS	1.66
Gly	7.70^b^	9.73^a^	7.84^b^	10.3^a^	5.69^c^	6.38^c^	6.06^c^	5.68^c^	***	***	NS	**	NS	NS	NS	1.24
His	1.02	0.60	1.83	1.19	2.02	1.68	2.16	1.93	*	***	*	NS	NS	NS	NS	0.77
Arg	4.40^b^	2.92^c^	5.09^ab^	5.91^a^	5.20^ab^	4.64^ab^	5.47^ab^	4.65^ab^	NS	NS	*	NS	NS	*	+	1.51
Thr	5.83	5.59	6.04	6.60	6.84	6.61	7.00	6.29	NS	**	NS	NS	NS	NS	NS	0.87
Ala	9.26	9.73	9.07	10.3	7.58	8.96	8.07	8.37	*	***	NS	NS	NS	NS	NS	1.40
Pro	4.98^a^	3.33^bc^	4.52^ab^	3.75^abc^	2.89^c^	3.02^c^	3.19^c^	3.11^bc^	+	***	NS	*	NS	NS	NS	1.18
Tyr	3.49	1.87	3.35	1.75	4.51	3.55	4.62	3.90	***	***	NS	NS	NS	NS	NS	0.97
Val	5.38	5.10	5.76	5.30	6.54	7.15	6.71	6.85	NS	***	NS	NS	NS	NS	NS	0.57
Ile	5.28^b^	4.92^bc^	5.00^bc^	4.46^c^	6.58^a^	6.94^a^	6.59^a^	6.78^a^	NS	***	NS	*	NS	NS	NS	0.58
Leu	5.86^b^	3.52^c^	6.08^b^	2.93^c^	6.77^ab^	8.24^a^	6.77^ab^	7.90^ab^	NS	***	NS	***	NS	NS	NS	1.89
Lys	7.70	5.61	7.47	5.93	8.90	8.30	9.18	8.64	+	**	NS	NS	NS	NS	NS	2.83
Phe	4.07	3.17	4.58	4.35	5.04	4.33	5.33	4.52	**	***	*	NS	NS	NS	NS	0.91

^1^ M, methanogenesis inhibition (control or 9, 10-anthraquinone); C, carbon source (cellulose or starch); N, nitrogen source (NH_4_^+^ or Try); RMSE, root mean square of the error; NH_4_^+^, ammonium; Try, trypticase. ^2^ DM, dry matter; N, nitrogen; AA, amino acids. ^3^ ND, mean was not determined; -, effect was not determined; NS, *p* > 0.10; +, 0.05 ≤ *p* < 0.10; *, 0.01 ≤ *p* < 0.05; **, 0.001 ≤ *p* < 0.01; ***, *p* < 0.001. ^4^ Unlike superscripts within a row indicate significant (*p* < 0.05) differences when interactions (*p* < 0.10) are present.

**Table 3:** Effects of the addition of 9, 10-anthraquinone to rumen batch cultures incubated with cellulose or starch as carbon and energy sources and ammonium or trypticase as sources of nitrogen on the net production of microbial amino acids.

Amino Acid	Treatments								M^2^	C	N	M × C	M × N	C × N	M × C × N	RMSE
	Cellulose				Starch											
	Net Synthesis^1^		Net Synthesis + Incorporation^1^		Net Synthesis		Net Synthesis + Incorporation									
	AQ-^2^	AQ+	AQ-	AQ+	AQ-	AQ+	AQ-	AQ+								
Asp (mg)	8.56^d4^	5.17^d^	10.5^cd^	5.28^d^	16.7^bc^	25.0^a^	18.7^ab^	16.2^bc^	NS^5^	***	NS	*	+	NS	NS	6.85
Glu (mg)	8.71^d^	3.99^d^	10.9^cd^	6.13^d^	17.7^bc^	27.9^a^	20.5^ab^	19.0^bc^	NS	***	NS	*	NS	NS	NS	7.70
Ser (mg)	3.27^c^	2.54^c^	4.46^bc^	2.97^c^	6.95^b^	10.9^a^	7.67^ab^	7.60^ab^	NS	***	NS	NS	NS	NS	NS	3.96
Gly (mg)	4.65^bc^	2.74^c^	5.50^bc^	3.97^c^	7.39^bc^	15.0^a^	9.49^b^	8.12^bc^	NS	***	NS	+	NS	NS	+	5.47
His (mg)	1.51^bc^	0.38^c^	1.37^bc^	0.49^c^	2.57^ab^	3.53^a^	3.27^a^	2.59^ab^	NS	***	NS	+	NS	NS	NS	1.34
Arg (mg)	2.56^de^	1.02^e^	3.86^cd^	1.88^de^	6.57^b^	9.53^a^	8.31^ab^	6.26^bc^	NS	***	NS	+	*	NS	+	2.38
Thr (mg)	3.47^d^	1.65^d^	4.57^cd^	2.48^d^	8.68^b^	14.4^a^	10.7^ab^	8.66^bc^	NS	***	NS	+	+	NS	+	3.98
Ala (mg)	5.56^cd^	2.69^d^	6.74^bcd^	3.97^cd^	10.2^bc^	20.8^a^	13.1^b^	11.0^bc^	NS	***	NS	*	+	NS	+	6.98
Pro (mg)	3.06^cde^	1.21^e^	3.47^bcd^	1.45^de^	3.85^bc^	6.61^a^	5.13^ab^	4.07^bc^	NS	***	NS	*	+	NS	+	2.14
Tyr (mg)	2.03^bc^	0.62^c^	2.49^b^	0.79^bc^	5.56^a^	6.98^a^	6.90^a^	5.23^a^	NS	***	NS	NS	+	NS	NS	1.83
Val (mg)	3.17^d^	1.42^d^	4.37^cd^	2.08^d^	8.47^bc^	15.6^a^	10.7^b^	9.08^bc^	NS	***	NS	*	*	NS	+	4.34
Ile (mg)	3.07^c^	1.32^c^	3.84^c^	1.74^c^	8.41^b^	14.8^a^	10.5^b^	9.04^b^	NS	***	NS	*	*	NS	+	3.81
Leu (mg)	3.28^c^	0.86^c^	4.70^c^	1.12^c^	9.40^b^	17.1^a^	11.2^b^	10.6^b^	NS	***	NS	**	+	NS	NS	7.09
Lys (mg)	4.70^c^	1.95^c^	5.81^c^	3.17^c^	11.4^b^	17.5^a^	14.6^ab^	11.4^b^	NS	***	NS	+	+	NS	*	4.61
Phe (mg)	2.39^cd^	0.97^d^	3.48^c^	1.52^cd^	6.22^b^	8.93^a^	8.14^ab^	6.10^b^	NS	***	NS	+	*	NS	+	2.19
Total AA^3^ (mg)	59.1^d^	28.5^d^	76.7^cd^	38.7^d^	130^bc^	215^a^	159^ab^	133^bc^	NS	***	NS	*	+	NS	+	59.0

^1^ Net synthesis of AA, Ammonium (NH_4_^+^) treatments; Net synthesis + incorporation of AA, Trypticase (Try) treatments (see Materials and Methods). ^2^ M, methanogenesis inhibition (Control or 9, 10-Anthraquinone); C, carbon source (Cellulose or Starch); N, nitrogen source (NH_4_^+^ or Try); RMSE, root mean square of the error. ^3^ AA, amino acids. ^4^ Unlike superscripts within a row indicate significant (*p* < 0.05) differences when interactions (*p* > 0.10) are present. ^5^ NS, *p* > 0.10; +, 0.05 ≤ *p* < 0.10; *, 0.01 ≤ *p* < 0.05; **, 0.001 ≤ *p* < 0.01; ***, *p* < 0.001.

**Table 4:** Effects of the addition of 9, 10-anthraquinone to rumen batch cultures incubated with cellulose or starch as carbon and energy sources and ammonium or trypticase as sources of nitrogen on the metabolic hydrogen balance.

	Treatments								M	C	N	M × C	M × N	C × N	M × C × N	RMSE
	Cellulose				Starch											
	NH_4_^+1^		Try		NH_4_^+^		Try									
	AQ-	AQ+	AQ-	AQ+	AQ-	AQ+	AQ-	AQ+								
**VFA and gases**																
**[2H] produced**																
Acetate (mmol)	9.82^e3^	6.30^f^	13.0^d^	9.78^e^	16.4^bc^	16.3^c^	20.9^a^	18.8^ab^	***^4^	***	***	+	NS	NS	NS	2.55
Propionate (mmol)	2.63^de^	0.95^f^	3.07^d^	1.63^ef^	6.14^b^	4.66^c^	8.06^a^	5.82^b^	***	***	***	NS	NS	+	NS	1.16
Butyrate (mmol)	4.10^d^	2.17^d^	4.66^d^	4.00^d^	23.2^c^	31.4^ab^	28.6^bc^	37.0^a^	*	***	*	**	NS	NS	NS	6.11
Valerate (mmol)	0.40^c^	0.38^c^	1.14^ab^	1.17^a^	0.45^c^	0.32^d^	1.00^b^	0.40^cd^	***	***	***	***	*	***	***	0.15
Total *[2H]_produced_*^2^ (mmol)	17.0^de^	9.80^e^	21.9^d^	16.6^de^	46.2^c^	52.6^bc^	58.5^ab^	62.1^a^	NS	***	***	*	NS	NS	NS	9.49
**[2H] incorporated**																
Propionate (mmol)	5.27^de^	1.91^f^	6.14^d^	3.26^ef^	12.3^b^	9.32^c^	16.1^a^	11.7^b^	***	***	***	NS	NS	+	NS	2.32
Butyrate (mmol)	2.05^d^	1.08^d^	2.33^d^	2.00^d^	11.6^c^	15.7^ab^	14.3^bc^	18.5^a^	*	***	*	**	NS	NS	NS	3.05
Valerate (mmol)	0.54^c^	0.50^c^	1.52^ab^	1.56^a^	0.61^c^	0.43^c^	1.33^b^	0.54^c^	***	***	***	***	**	***	***	0.20
CH_4_ (mmol)	0.94^cd^	0.42^d^	1.64^bc^	0.34^d^	2.29^b^	0.90^cd^	4.02^a^	0.97^cd^	***	***	*	*	*	NS	NS	1.01
H_2_ (mmol)	0.022^e^	0.044^e^	0.024^e^	0.074^e^	1.00^c^	1.20^b^	0.80^d^	1.61^a^	***	***	NS	***	***	NS	***	0.16
Total *[2H]_incorporated_* (mmol)	8.82^cd^	3.94^d^	11.6^c^	7.23^cd^	27.8^b^	27.6^b^	36.6^a^	33.3^a^	*	***	***	NS	NS	+	NS	5.16
*[2H]_recovery_* (%*[2H]_produced_*)	51.7^b^	39.4^d^	52.9^b^	43.2^c^	60.4^a^	52.9^b^	62.9^a^	53.9^b^	***	***	**	+	NS	NS	NS	3.12
**Amino acids: minimum net [2H] incorporation**																
*[2H]_produced_* (mmol)	1.30^bc^	0.61^c^	ND	ND	2.26^b^	4.86^a^	ND	ND	+	***	-	**	-	-	-	1.45
*[2H]_incorporated_* (mmol)	0.84^bc^	0.38^c^	ND	ND	1.51^b^	3.23^a^	ND	ND	+	***	-	**	-	-	-	0.96
*∆[2H]_incorporated_* (mmol)	−0.47^a^	−0.23^a^	ND	ND	−0.74^a^	−1.63^b^	ND	ND	+	***	-	**	-	-	-	0.50
**Amino acids: maximum net [2H] incorporation**																
*[2H]_produced_* (mmol)	0.81^bc^	0.43^c^	ND	ND	1.41^b^	2.97^a^	ND	ND	+	***	-	**	-	-	-	0.91
*[2H]_incorporated_* (mmol)	1.27^bc^	0.56^c^	ND	ND	2.23^b^	4.76^a^	ND	ND	+	***	-	**	-	-	-	1.41
*∆[2H]_incorporated_* (mmol)	0.46^bc^	0.15^c^	ND	ND	0.83^b^	1.81^a^	ND	ND	+	***	-	**	-	-	-	0.50
**Overall [2H] balance in VFA, gases and amino acids**																
Min *[2H]_recovery_* (%*[2H]_produced_*)	52.7^b^	40.6^c^	ND	ND	60.6^a^	53.3^b^	ND	ND	***	***	-	*	-	-	-	3.03
Max *[2H]_recovery_* (%*[2H]_produced_*)	56.6^b^	43.1^c^	ND	ND	63.4^a^	57.6^b^	ND	ND	***	***	-	*	-	-	-	4.00

^1^ NH_4_^+^, ammonium; Try, trypticase; AQ-, Control (non-inhibited methanogenesis) treatment; AQ+, 9, 10-Anthraquinone (methanogenesis inhibited) treatment; M, methanogenesis inhibition (Control or 9, 10-Anthraquinone); C, carbon source (Cellulose or Starch); RMSE, root mean square of the error; VFA, volatile fatty acids. ^2^ *[2H]_produced_*, pairs of reducing equivalents produced; *[2H]_incorporated_*, pairs of reducing equivalents incorporated; CH_4_, methane; H_2_, dihydrogen; *[2H]_recovery_* = percentage of pairs of reducing equivalents produced recovered in *[2H]_incorporated_*; *∆[2H]_incorporated_*, *[2H]_incorporated_* - *[2H]_produced_*; Min *[2H]_recovery_*, *[2H]_recovery_* calculated with minimal incorporation of [2H] into the synthesis of amino acids; Max *[2H]_recovery_*, *[2H]_recovery_* calculated with maximal incorporation of [2H] into the synthesis of amino acids. ^3^ Unlike superscripts within a row indicate significant (*p* < 0.05) differences when interactions (*p* < 0.10) are present. ^4^ NS, *p* > 0.10; +, 0.05 ≤ *p* < 0.10; *, 0.01 ≤ *p* < 0.05; **, 0.001 ≤ *p* < 0.01; ***, *p* < 0.001; ND, mean was not determined; -, effect was not determined.

## Supplementary Materials

The following are available online at https://www.mdpi.com/2076-2607/8/6/799/s1, Table S1: Composition of the ammonium and trypticase media, Table S2: Pairs of reducing equivalents ([2H]) produced or incorporated associated to the formation of volatile fatty acids and gases from glucose in rumen fermentation, Table S3. Pairs of reducing equivalents ([2H]) produced and incorporated associated to the formation of amino acids from glucose, carbon dioxide (CO_2_), volatile fatty acids (VFA) and ammonium (NH_4_^+^) in rumen fermentation, Figure S1: Evolution over time (*t*) of total gas production in Control or 9, 10-anthraquinone (AQ)-supplemented rumen batch cultures fermenting cellulose (C) or starch with ammonium (NH_4_^+^) or trypticase as nitrogen source. The random effects of the incubation (*I*) and bottle (*B*) are included in the model: Total gas (atm) = 0.14 (±0.0090; *p* < 0.001) – 0.33 (±0.0054; *p* < 0.001) cellulose – 0.0091 (±0.0052; *p* < 0.087) NH_4_^+^ + 0.0071 (±0.0054; *p* = 0.19) AQ + 0.010 (±0.00020; *p* < 0.001) *t*_(h)_ – 0.036 (±0.0054; *p* < 0.001) C × AQ – 0.0071 (±0.00020; *p* < 0.001) C × *t* – 0.00043 (±0.00019; *p* = 0.025) NH_4_^+^ × *t* – 0.00013 (±0.00020; *p* = 0.54) AQ × *t* – 0.00078 (±0.00020; *p* < 0.001) C × AQ × *t* + *I* (random; *p* = 0.53) + *B* (random; *p* = 0.18) + *error*; R^2^ = 0.94.
