# Supplementary material for: Inhibiting Methanogenesis Stimulated de novo Synthesis of Microbial Amino Acids in Mixed Rumen Batch Cultures Growing on Starch but not on Cellulose
Source: Microorganisms. 2020 May 26;8(6):799. doi: 10.3390/microorganisms8060799 (PMC7356843; doi:10.3390/microorganisms8060799)
Supplement: Supplementary file 1 [file microorganisms-08-00799-s001.pdf]

Table S1. Composition of the ammonium and trypticase media.

Component (mL or g/L)	Ingredient	Ammonia (NH_3) medium (g/L)	Trypticase (Trp) medium (g/L)
Buffer (245)	NH_4HCO_3	4.00	-
	Trypticase	-	8.25
	$NaHCO_3$	35.0	39.25
Macromineral solution (245)	Na_2HPO_4	5.70	5.70
	KH_2PO_4	6.20	6.20
	$MgSO_4.7H_2O$	0.60	0.60
Micromineral solution (0.1)	[a]		
Pfennings solution (10)	[b]		
Resazurin solution (1.0)	[c]		
Volatile fatty acids (VFA) solution (10)	[d]		
Yeast extract (0.50)	-		
Distilled water (490)	-		
Reducing solution (50)	[e]		

[a] In g/L: $CaCl_2.2H_2O$ = 132, $MnCl_2.4H_2O$ = 100, $CoCl_2.6H_2O$ = 10, $FeCl_3.6H_2O$ = 80. [b] In mg/L: Na_4EDTA = 500, $FeSO_4.7H_2O$ = 200, $MnCl_2.4H_2O$ = 200, $ZnSO_4.7H_2O$ = 10; H_3BO_3 = 30; $CoCl_2.6H_2O$ = 20, $CuCl_2.2H_2O$ = 1, $NiCl_2.6H_2O$ = 2, $NaMoO_4.2H_2O$ = 3. [c] 1 g/L. [d] Acetic acid = 17 mL, propionic acid = 6 mL, butyric acid = 4 mL, isobutyric acid = 1 mL, 2-methylbutyric acid = 1 mL, isovaleric acid = 1 mL, valeric acid = 1 mL, taken to 100 mL with distilled water and neutralized to pH 7 with 1 N NaOH. [e] Cysteine hydrochloride = 0.625 g, distilled water = 95 mL, 1 N NaOH = 4 mL, $Na_2S.9H_2O$ = 0.625 g.

Table S2. Pairs of reducing equivalents ([2H]) produced or incorporated associated to the formation of volatile fatty acids and gases from glucose in rumen fermentation.

Metabolite	Overall stoichiometry	$[2H]_{produced}$ (mol/mol metabolite)	$[2H]_{incorporated}$ (mol/mol metabolite)
Acetate	$C_6H_{12}O_6 + 2\ H_2O \rightarrow 2\ CH_3COO^- + 2\ H^+ + 2\ CO_2 + 4\ [2H]$	2	0
Propionate	$C_6H_{12}O_6 + 2\ [2H] \rightarrow 2\ CH_3CH_2COO^- + 2\ H^+ + 2\ H_2O$	1	2
Butyrate	$C_6H_{12}O_6 \rightarrow CH_3CH_2CH_2COO^- + H^+ + 2\ CO_2 + 2\ [2H]$	4	2
Valerate	$C_6H_{12}O_6 + [2H] \rightarrow CH_3CH_2CH_2CH_2COO^- + H^+ + CO_2 + 2\ H_2O$	3	4
Methane	$CO_2 + 4\ [2H] \rightarrow CH_4 + 2\ H_2O$	0	4
Hydrogen	$[2H] \rightarrow H_2$	0	1

Table S3. Pairs of reducing equivalents ([2H]) produced and incorporated associated to the formation of amino acids from glucose, carbon dioxide (CO_2), volatile fatty acids (VFA) and ammonium (NH_4^+) in rumen fermentation.

Equation	Amino acid	Overall stoichiometry of synthesis	[2H] produced (mol/mol amino acid)	[2H] incorporated (mol/mol amino acid)	Δ[2H] [(mol incorporated – mol produced)/mol amino acid]	Comment
1	Glu	$C_6H_{12}O_6$ + 4 CO_2 + 2 NH_4^+ + 6 [2H] → 2 $COO^-CH_2CH_2CHNH_3^+COO^-$ + 4 H^+ + 6 H_2O	1	4	3	α-ketoglutarate produced by reverse tricarboxylic acid cycle (TCA)
2	Glu	$C_6H_{12}O_6$ + NH_4^+ + CO_2 → $COO^-CH_2CH_2CHNH_3^+COO^-$ + 2 H^+ + 2 CO_2 + 3 [2H]	4	1	-3	α-ketoglutarate produced by forward TCA
3	Asp	$C_6H_{12}O_6$ + 2 NH_4^+ + 2 CO_2 → 2 $COO^-CH_2CHNH_3^+COO^-$ + 4 H^+ + 2 H_2O	1	1	0	Oxaloacetate formed from pyruvate or phosphoenolpyruvate carboxylation
4	Ala	$C_6H_{12}O_6$ + 2 NH_4^+ → 2 $CH_3CHNH_3^+COO^-$ + 2 H^+ + 2 H_20	1	1	0	Pyruvate formed in glycolysis
5	Ala	CH_3COO^- + CO_2 + NH_4^+ + 2 [2H] → $CH_3CHNH_3^+COO^-$ + 2 H_2O	0	2	2	Pyruvate formed by reductive carboxylation of acetate added to the medium
6	Pro	$C_6H_{12}O_6$ + 4 CO_2 + 2 NH_4^+ + 10 [2H] → 2 $(CH_2)_3NHCHCOO^-$ + 4 H^+ + 10 H_2O	1	6	5	Glu formed through Eq. 1
7	Pro	$C_6H_{12}O_6$ + NH_4^+ → $(CH_2)_3NHCHCOO^-$ + CO_2 + 2 H_2O + [2H] + 2 H^+	4	3	-1	Glu formed through Eq. 2
8	Arg	$C_6H_{12}O_6$ + 8 NH_4^+ + 6 CO_2 + 10 [2H] → 2 $H_2N^+CNH_2NH(CH_2)_3CHNH_3^+COO^-$ + 6 H^+ + 14 H_2O	2	7	5	Glu formed through Eq. 1

9	Arg	$C_6H_{12}O_6 + 4\ NH_4^+ \rightarrow H_2N^+CNH_2NH(CH_2)_3CHNH_3^+COO^- + 3\ H^+ + 4\ H_2O + [2H]$	5	4	-1	Glu formed through Eq. 2
10	Ser	$C_6H_{12}O_6 + 2\ NH_4^+ \rightarrow 2\ HOCH_2CHNH_3^+COO^- + 2\ H^+ + 2\ [2H]$	2	1	-1	From 3-phosphoglycerate
11	Gly	$C_6H_{12}O_6 + 2\ NH_4^+ + 2\ H_2O \rightarrow 2\ CH_2NH_3^+COO^- + 2\ H^+ + 2\ CO_2 + 6\ [2H]$	4	1	-3	From ser. One [2H] pair released in formate from regeneration of methylene-THF to THF
12	Lys	$2\ C_6H_{12}O_6 + 4\ NH_4^+ + 4\ [2H] \rightarrow 2\ CH_2NH_3^+(CH_2)_3CHNH_3^+COO^- + 8\ H_2O + 2\ H^+$	2	4	2	From asp
13	Thr	$C_6H_{12}O_6 + 2\ NH_4^+ + 2\ CO_2 + 4\ [2H] \rightarrow 2\ CH_3CHOHCHNH_3^+COO^- + 2\ H^+ + 4\ H_2O$	1	3	2	From asp
14	Val	$C_6H_{12}O_6 + NH_4^+ \rightarrow CH_3CHCH_3CHNH_3^+COO^- + H^+ + CO_2 + 2\ H_2O$	2	2	0	From glucose
15	Val	CH_3CHCH_3COO- $+ CO_2 + NH_4^+ + 2\ [2H] \rightarrow CH_3CHCH_3CHNH_3^+COO^- + 2\ H_2O$	0	2	2	Reductive carboxylation of isobutyrate followed by amination
16	Leu	$3\ C_6H_{12}O_6 + 2\ NH_4^+ \rightarrow 2\ (CH_3)_2CHCH_2CHNH_3^+COO^- + 6\ CO_2 + 2\ H_2O + 6\ [2H] + 2\ H^+$	5	2	-3	Glucose as sole carbon source
17	Leu	$C_6H_{12}O_6 + CH_3COO^- + NH_4^+ \rightarrow (CH_3)_2CHCH_2CHNH_3^+COO^- + 2\ CO_2 + 2\ H_2O + [2H]$	3	2	-1	Carbons 1 and 2 contributed by preformed acetate
18	Leu	$CH_3CHCH_3CH_2COO^- + CO_2 + NH_4^+ + 2\ [2H] \rightarrow (CH_3)_2CHCH_2CHNH_3^+COO^- + 2\ H_2O$	0	2	2	Reductive carboxylation of isovalerate followed by amination
19	Ile	$C_6H_{12}O_6 + NH_4^+ + 3\ [2H] \rightarrow CH_3CH_2CHCH_3CHNH_3^+COO^- + 4\ H_2O + H^+$	2	5	3	Glucose as sole carbon source
20	Ile	$CH_3CH_2CHCH_3COO$- $+ CO_2 + NH_4^+ + 2\ [2H] \rightarrow CH_3CH_2CHCH_3CHNH_3^+COO^- + 2\ H_2O$	0	2	2	Reductive carboxylation of 2-methylbutyrate followed by amination

21	Tyr	2 $C_6H_{12}O_6$ + NH_4^+ → $C_9H_{11}NO_3$ + 3 CO_2 + 3 H_2O + H^+ + 5 [2H]	7	2	-5	Shikimate cycle. Erythrose-4-phosphate from pentose cycle
22	Phe	2 $C_6H_{12}O_6$ + NH_4^+ → $C_9H_{11}NO_2$ + 3 CO_2 + 4 H_2O + H^+ + 4 [2H]	6	2	-4	Shikimate cycle. Erythrose-4-phosphate from pentose cycle
23	His	$C_6H_{12}O_6$ + H_2O + ATP + 2 NH_4^+ → $C_6H_9N_3O_2$ + 5-aminoimidazole-4-caboxamide ribonucleotide + CO_2 + 3 [2H]	3	0	-3	Phosphoribosyl pyrophosphate from pentose cycle.

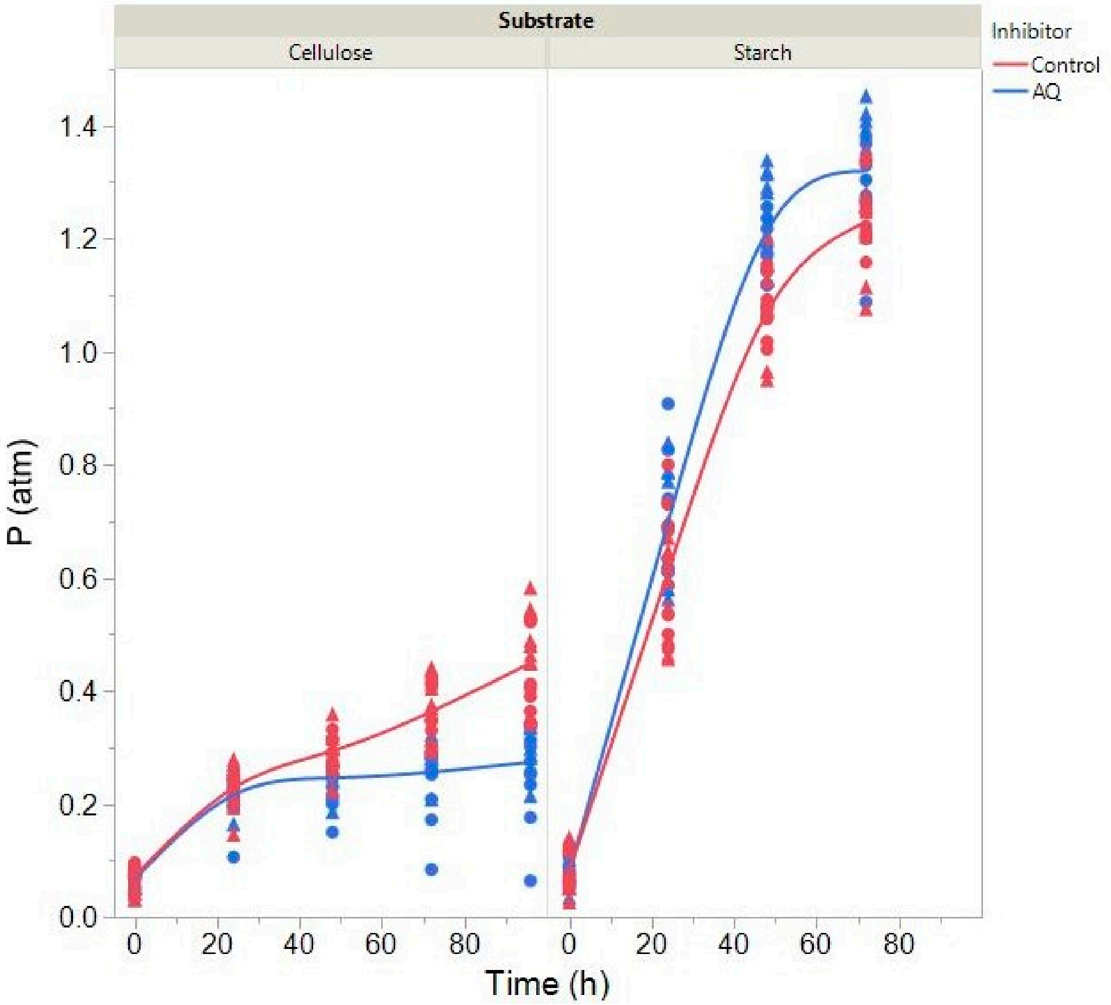

Figure S1. Evolution over time (*t*) of total gas production in Control or 9, 10-anthraquinone (AQ)-supplemented rumen batch cultures fermenting cellulose (C) or starch with ammonium (NH_4^+) or trypticase as nitrogen source. The random effects of the incubation (*I*) and bottle (*B*) are included in the model: Total gas (atm) = 0.14 (±0.0090; $p < 0.001$) − 0.33 (±0.0054; $p < 0.001$) cellulose − 0.0091 (±0.0052; $p < 0.087$) NH_4^+ + 0.0071 (±0.0054; $p = 0.19$) AQ + 0.010 (±0.00020; $p < 0.001$) $t_{(h)}$ − 0.036 (±0.0054; $p < 0.001$) C × AQ − 0.0071 (±0.00020; $p < 0.001$) C × t − 0.00043 (±0.00019; $p = 0.025$) $NH_4^+ \times t$ − 0.00013 (±0.00020; $p = 0.54$) AQ × t − 0.00078 (±0.00020; $p < 0.001$) C × AQ × t + I (random; $p = 0.53$) + B (random; $p = 0.18$) + *residual*; $R^2 = 0.94$.
